# Effects of One-Step Hot Oil Treatment on the Physical, Mechanical, and Surface Properties of Bamboo Scrimber

**DOI:** 10.3390/molecules25194488

**Published:** 2020-09-30

**Authors:** Zhurun Yuan, Xinwu Wu, Xinzhou Wang, Xi Zhang, Tiancheng Yuan, Xianmiao Liu, Yanjun Li

**Affiliations:** 1 College of Materials Science and Engineering, Nanjing Forestry University, Nanjing 210037, China; yuanzr0311@163.com (Z.Y.); 15717414150@163.com (X.W.); xzwang@njfu.edu.cn (X.W.); m15720613205@163.com (X.Z.); ytc_njfu@163.com (T.Y.); 2 International Center for Bamboo and Rattan, Beijing 100102, China; 3 Jiangsu Co-Innovation Center of Efficient Processing and Utilization of Forest Resources, Nanjing 210037, China

**Keywords:** hot oil treatment, bamboo scrimber, mechanical properties, dimensional stability

## Abstract

Bamboo scrimber is a new type of bamboo-based panel that is prone to be affected by biological and service environments under outdoor conditions. In this paper, the physical and mechanical performance and the microchemical and surface properties of untreated and hot-oil-treated bamboo scrimber were analyzed to illustrate the processing mechanism of scrimber. Methyl silicone oil treatment was carried out at 120, 140, and 160 °C for 2, 4, and 6 h. The density, mechanical properties, air-dried moisture content, surface morphology, chemical structure, swelling properties, color, and contact angle of the bamboo scrimber were analyzed to evaluate the treatment effectiveness. Observation of the environmental-scanning electron microscope indicated that the glue layer of the bamboo scrimber was not significantly damaged after hot oil treatment. At low temperatures, the mechanical properties did not change significantly. Infrared-spectrum analysis showed a significant decrease in mechanical properties at higher temperatures and longer treatment time for the degradation of hemicellulose. The contact angle test and swelling properties test showed that the hot oil treatment improved the dimensional stability and reduced the wettability on the surface of the bamboo scrimber. The above analysis results show that the treatment at 140 °C for 2 h is most effective.

## 1. Introduction

As a material with a short growth cycle, bamboo resources are widely distributed in the Asia-pacific region, the Americas, and Africa. Bamboo is a light and high-strength material often used for building materials, musical instruments, stationery, farm tools, and crafts [1,2]. The efficient use of bamboo can ensure the adequate supply of world fiber products.

Scholars from various countries have extensively studied the use of bamboo. Bamboo-based products such as bamboo curtain composite, plybamboo, and laminated bamboo lumber for structural use have been successively developed [3,4]. Bamboo scrimber is a new type of bamboo-based composite that is manufactured by hot or cold pressure on the bamboo bundle. The development of bamboo scrimber not only improves the utilization of bamboo, but also provides a good option for structural engineering due to its excellent performance [5,6]. Indeed, numerous studies have shown that bamboo scrimber has good application prospects in structural buildings. However, bamboo is rich in sugar and has abundant starch, which leads to its high moisture absorption and poor biological durability. Therefore, bamboo scrimber absorbs water easily and can form cracking defects under outdoor conditions, which greatly reduces the safety coefficient of the bamboo scrimber in engineering utilization [5].

Methods of modifying the bamboo mainly include chemical methods and physical methods. The chemical methods generally include the addition of fungicides and flame retardants, while the physical methods include types of heat treatments [7,8]. Heat treatment is a physical modification technology in which biomass materials are treated in a high-temperature, low-oxygen, or even anaerobic environment that greatly improves the dimensional stability, anticorrosion, and mildew resistance of bamboo scrimber products [9]. In the literature, the bamboo bundles were pre-heated to manufacture bamboo scrimber. The resulting products had good water-absorption, thickness, and width-expansion properties, and resistance to fungal corrosion. They were suitable for outdoor landscaping, indoor and outdoor decoration, garden furniture, etc. [10,11]. Hot oil is also an excellent heat-conducting medium, which has good application prospects in the treatment of biomass materials. Some studies on the modification of bamboo and wood with hot oil have found that hot oil treatment can improve dimensional stability and resistance to mold [12,13]. Moreover, hot oil can form an oil film on the surface of the bamboo, which has a certain protective effect on biomass materials. However, hot oil treatment is relatively high-cost and may adversely affect the surface of the material, which is not conducive to industrial production [13]. Heat treatment of the bamboo unit is not only cumbersome, but also affects the bonding performance of the bamboo bundle and the physical and mechanical properties of the product. Therefore, a simple one-step processing technology has become a research hotspot.

In this paper, methyl silicone oil is used as a medium to directly treat the bamboo scrimber board. The effects of the temperature and duration of the heat treatment on the physical, mechanical, and surface properties of bamboo scrimber were investigated to find a one-step method to improve the performance of bamboo scrimber.

## 2. Results and Discussion

### 2.1. Microstructure

**Figure 1:**
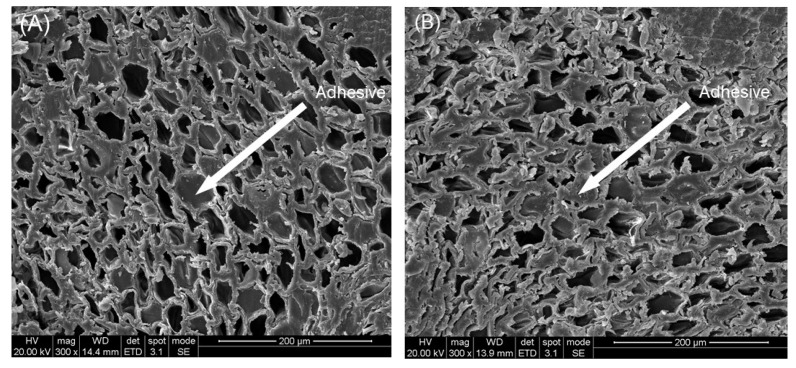
(**A**) The micromorphology of the bamboo scrimber before hot oil treatment; (**B**) the micromorphology of the bamboo scrimber after hot oil treatment.

The microscopic morphology of the bamboo scrimber before and after treatment is shown in Figure 1. Although the bamboo parenchyma cells became more distorted after the hot oil treatment, the cell wall was not significantly damaged. The glue layer was able to be clearly observed, and the glue existed in the cell cavity, indicating that the hot oil treatment does not damage the cell wall structure of bamboo parenchyma cells and has little effect on the glue layer. This suggests that oil treatment has no obvious negative influence on the bonding properties of bamboo scrimber [14,15].

### 2.2. FTIR Analysis

**Figure 2:**
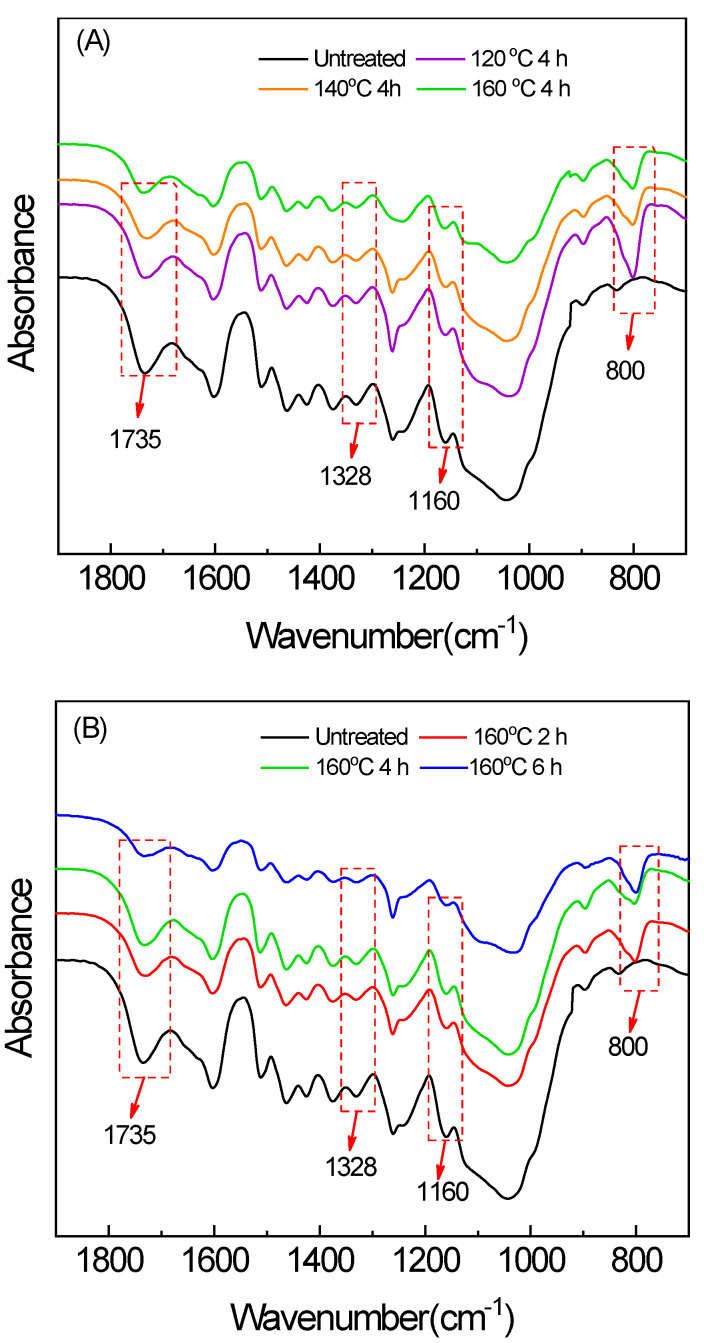
(**A**) FTIR spectra of the bamboo scrimber after hot oil treatment (temperature is variable); (**B**) FTIR spectra of the bamboo scrimber after hot oil treatment (time is variable).

The FTIR spectra of the untreated and treated samples are presented in Figure 2. The differences in the spectra of the untreated and treated samples at different treatment conditions were evaluated to understand the structural changes that occur within the polymeric structure of the bamboo during hot oil treatment. The shape of infrared spectrum did not change obviously after treatment, indicating that the bamboo scrimber did not undergo drastic chemical changes after treatment. A new band at 800 cm^−1^ found in the treated samples confirmed the presence of stretching vibration Si-C in Si-CH3 of methyl silicone oil [16]. There was a peak at 1736 cm^−1^ due to absorption of the carbonyl group, in which the intensity decreased. Absorption decreased significantly at the treatment condition of 160 °C and 6 h resulting from the degradation of hemicellulose under that condition. The absorption peak at 1159 cm^−1^ (C-O-C stretching vibration) and 1318 cm^−1^ (O-H stretching vibration) also decreased, confirming that the hemicellulose was dramatically degraded [17]. Lignin and phenolic resins are more difficult to decompose at lower temperatures [18]. Further cross-linking of phenolic resin can take place in the bamboo scrimber [19].

### 2.3. Physical Properties

**Figure 3:**
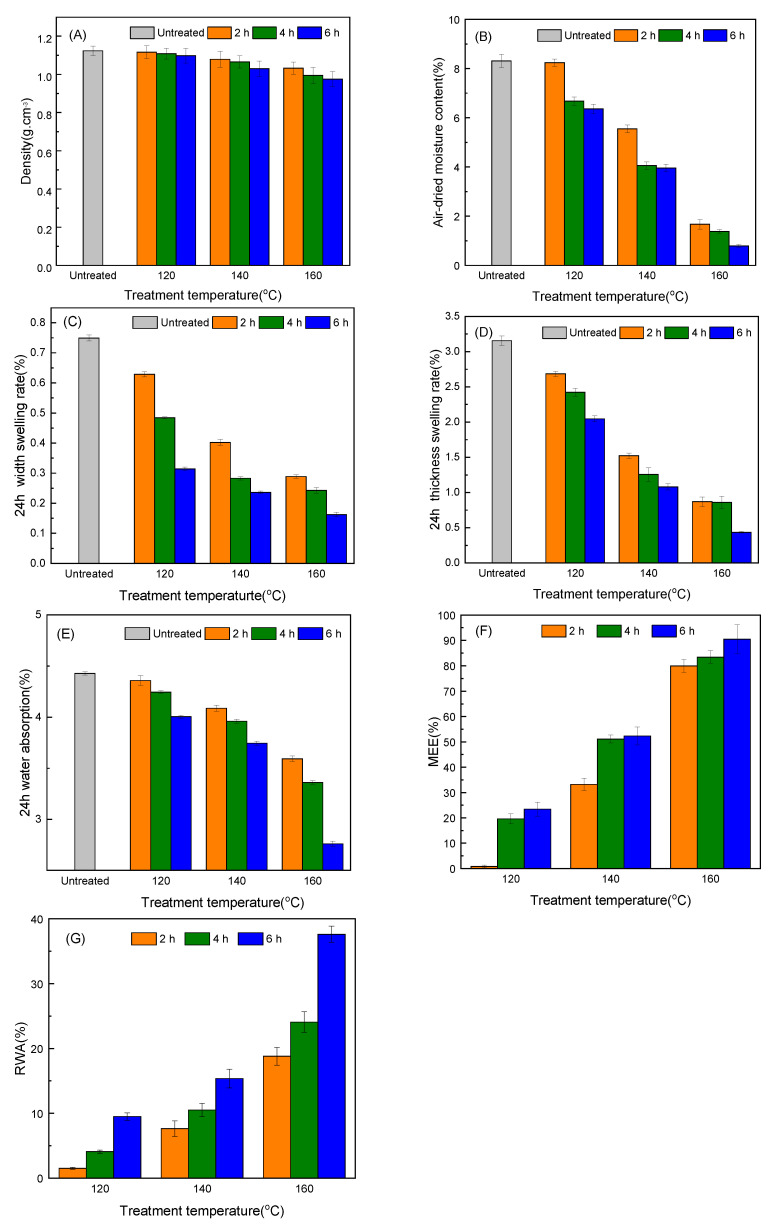
(**A**) The density of bamboo scrimber, (**B**) air-dried moisture content, (**C**) the 24 h width swelling rate, (**D**) the 24 h thickness swelling rate, (**E**) the 24 h water absorption, (**F**) moisture excluding efficiency (MEE), and (**G**) resistance to water absorption (RWA). Error Bars Represents the Standard Deviation of the Data.

The density change of the treated and untreated samples is shown in Figure 3A. The results (<0.01) of *p*-test of the air-dry density indicate that time and temperature had an extremely significant impact on density. As the temperature of the oil and the treatment time increased, the density gradually decreased, but the falling range was moderate. Temperature had a greater influence on the density than time. Compared with the untreated samples, the degree of decline was 12.39% and 14.16% at 160 °C for 4 h and 6 h, respectively. Air-dried moisture content for bamboo scrimber samples at different temperatures is shown in Figure 3B, and time and temperature had an extremely significant impact according to the result (<0.01) of *p*-test. Compared with the untreated samples (8.31%), the air-dried moisture content of the treated samples showed a downward trend, with a decreasing rate of 0.84~90.49%. The moisture excluding efficiency (MEE) of the treated samples are shown in Figure 3F, which indicates an upward trend, showing that hot oil treatment reduced the moisture absorption capacity of the bamboo scrimber. The influence of temperature on moisture content was more significant than that of time.

The water absorption expansion rate and water absorption rate of the bamboo scrimber samples are shown in Figure 3C–E. The *p*-test results showed that temperature (<0.01) had an extremely significant impact on 24 h thickness swelling rate, 24 h width swelling rate, and 24 h water absorption rate, and that the impact of time (<0.05) was significant. With the increase of the temperature and treatment time, the 24 h thickness swelling rate, 24 h width swelling rate, and 24 h water absorption rate of the samples decreased gradually, reaching the maximum rates of decline at 160 °C for 6 h treatment process, which were 86.07%, 78.67%, and 37.70%, respectively. The resistance to water absorption (RWA) of the treated samples is shown in Figure 3G, which indicates an upward trend and shows that hot oil treatment reduced the water absorption capacity of the bamboo scrimber. The hydrophilic groups of the hemicellulose in the bamboo and the free hydroxyl groups in the cellulose absorbed moisture, resulting in the stronger moisture absorption of the bamboo scrimber [20]. During the treatment process, the polysaccharides in hemicellulose were decomposed and polymerized to form water-insoluble polymers. At the same time, the free hydroxyl group in cellulose formed hydrogen bonds [17]. After treatment, the hydrogen bonds between cellulose molecular chains recombined, resulting in a decrease in the number of free hydroxyl groups and a decrease in the hygroscopic properties of the bamboo scrimber. Non-polar oil molecules attached to the cell wall may further prevent the entry of water after treatment [21]. Hot oil treatment improves the dimensional stability of the products.

### 2.4. Mechanical Properties

**Figure 4:**
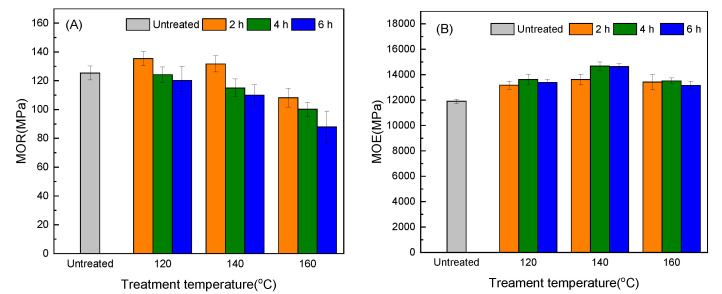
(**A**) The modulus of rupture (MOR) of bamboo scrimber after hot oil treatment; (**B**) the modulus of elasticity (MOE) of bamboo scrimber after hot oil treatment. Error bars represents the standard deviation of the data.

The influence of hot oil treatment on the modulus of rupture (MOR) of the bamboo scrimber is shown in Figure 4A. Temperature and time had a significant impact on the MOR according to the results (<0.05) of *p*-test. The MOR gradually decreased with the extension of the treatment time and the increase of the treatment temperature. At 120 °C and 140 °C for 2 h, the MOR increased by 8.00% and 5.08%, respectively, due to the decrease of air-dried moisture content [22]. However, the MOR decreased as the treatment time increased because of the degradation of hemicellulose, but the change was not obvious as compared to the control. The treatment time is extended mainly to extend the time of the internal reaction of the molecule and store more intermolecular activation energy [23], causing the hemicellulose to continue to degrade. When the temperature was 160 °C and the treatment time was 2 h and 4 h, the MOR decreased for the continuous degradation of hemicellulose. When the treatment time was 6 h, the MOR decreased by 29.90% due to the massive degradation of hemicellulose.

The effect of heat-treatment time and temperature on the modulus of elasticity (MOE) of bamboo scrimber is shown in Figure 4B. Temperature and time had a significant impact on MOE according to the results (<0.05) of *p*-test. After hot oil treatment, the elastic modulus of bamboo scrimber primarily increased as the air-dried moisture content declined. At 140 °C and 2 h, the MOE reached the maximum value of 23.26%, however, it showed a decreasing trend when the temperature reached 160 °C. This was due to the abundant decomposition of hemicellulose, though the value was still larger than that of the untreated samples. The glue layer of the bamboo scrimber was not damaged after the hot oil treatment. Combined with the electron microscope image (Figure 2), this indicates that the hot oil treatment has unobvious effects on the mechanical properties of the bamboo scrimber.

### 2.5. Surface Color Changes

**Figure 5:**
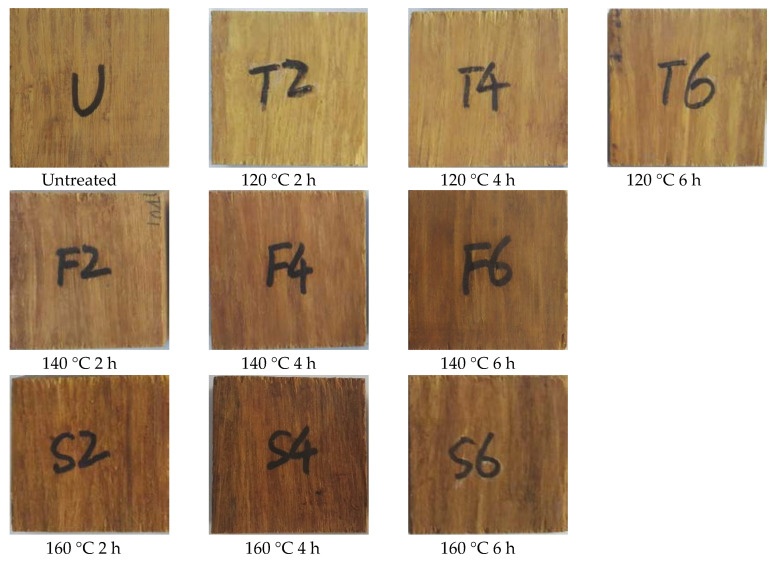
Digital photographs of bamboo scrimber before and after hot oil treatment.

Digital photographs of the bamboo scrimber after hot oil treatment are shown in Figure 5. The color of untreated bamboo scrimber was dark yellow and became darker after treatment. As the treatment temperature and time increased, the color changed to brown. The color of bamboo scrimber after treatment is darker than that of untreated group [24].

Table 1 shows the change in color index of the bamboo scrimber after hot oil treatment at different temperatures and times. After hot oil treatment, ΔE* values increased significantly, and L* values decreased with the increase of temperature and time, indicating that the surface color of the bamboo scrimber became darker after treatment. This was caused by (1) the degradation of the oxygen-containing groups of hemicelluloses such as acetyl and carboxyl groups, and (2) the increase in the relative content of lignin [25]. The increasing values of a* indicate a tendency of the sample surface to become reddish. However, as the temperature and time continued to increase, the change of a* tended to be flat, indicating that this influence was limited.

### 2.6. Wettability

**Figure 6:**
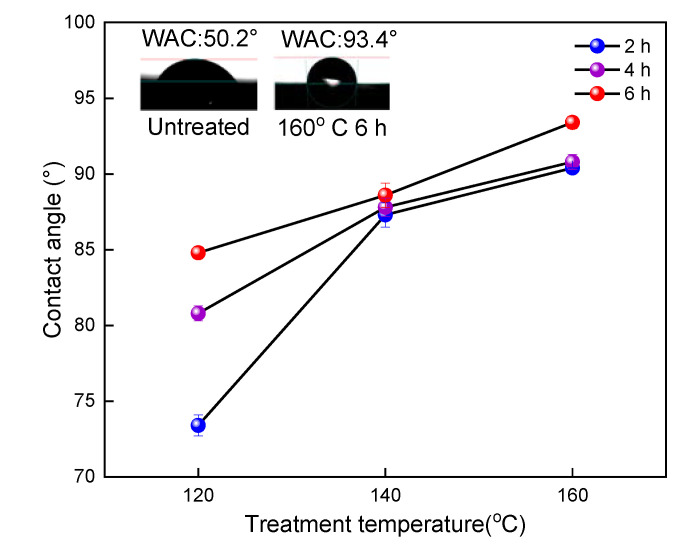
Contact angle of bamboo scrimber after hot oil treatment.

The contact angle of each group of samples is shown in Figure 6. The contact angle is the most intuitive parameter that indicates the surface wettability of the bamboo scrimber timber. Larger contact angle means less wettability and shows the greater hydrophobicity of the bamboo scrimber [26]. The contact angle of the untreated group was 50.2°, and that of the 160 °C and 6 h treatment group was 93.4°. It can be seen that as the temperature of oil increases, the contact angle of bamboo scrimber tends to increase and the influence of temperature on the contact angle is significantly greater than the influence of time. The increase in the contact angle of the bamboo scrimber was due to the dehydration of the hydrogen bonds of the cellulose into ether and hemicellulose pyrolysis after treatment, which reduced the hydrophilicity of the bamboo scrimber [27,28]. Moreover, the formation of oil film on the surface of the bamboo scrimber also has a great influence on hydrophobicity.

## 3. Materials and Methods

### 3.1. Materials

Outdoor bamboo scrimber board (Zhejiang Yongyu Bamboo Industry Co., Ltd., Huzhou, China, cold-pressed by bamboo bundles with phenolic adhesive; density: 1.1 g/cm^3^, dimension: 1980 (Length) × 140 (Width) × 10 (Thickness) mm^3^. Methyl silicone oil (Dow Corning imported silicone oil, density: 0.965 g/cm^3^, flash point: 300 °C, viscosity: 100 cs). Phenolic resin (Nanjing Tai er Chemical Co., Ltd., Nanjing, China, solid content: 43%, viscosity at 25 °C: 160 cps, pH: 13.3).

### 3.2. Methods

#### 3.2.1. Hot Oil Treatment

**Figure 7:**
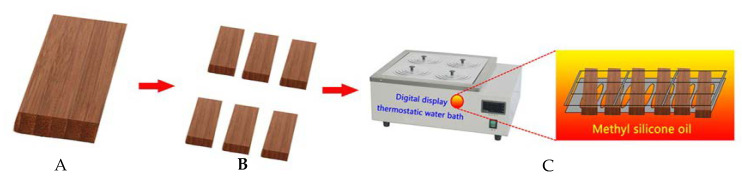
Schematic diagram of hot oil treatment. (**A**) Bamboo scrimber. (**B**) Saw cutting. (**C**) Hot oil treatment.

The bamboo scrimber with an initial moisture content of about 10% was sawed into samples with the dimensions of 460 (Length) × 140 (Width) × 10 (Thickness) mm^3^. Treatment was carried out in oil bath (HH-6S, Jintan Science Analysis Instrument Co., LTD., Jintan, China) containing methyl silicone oil with programmable controller. Methyl silicone oil served as a medium of heat transfer. As shown in Figure 7, samples were transferred and immersed in the oil bath with an iron frame. The treatment temperatures were 120 °C, 140 °C, and 160 °C, and the treatment times were 2 h, 4 h, and 6 h. The temperature was firstly raised to 120 °C at the speed of 15~20 °C/h and then raised to the specified temperatures by about 10~15 °C/h. After the treatment, the temperature was reduced to 60 °C.

#### 3.2.2. Morphological Characterization

The untreated and treated bamboo scrimber samples were cut into small specimens with the dimensions of 5 (thickness, perpendicular to fiber direction) × 5 (width, perpendicular to fiber direction) × 10 (length, parallel to fiber direction) mm^3^, and the microstructure of the cross-section of the bamboo scrimber was observed by using an environmental-scanning electron microscope (Quanta 200, FEI Company, Hillsboro, OR, USA) after sputter-coating with gold.

#### 3.2.3. Fourier Transform Infrared Spectroscopy

Fourier Transform Infrared Spectroscopy (FTIR) spectra were recorded as KBr disks on an FTIR spectrometer (VERTEX 80V, BRUKER, Ettlingen, Germany) at a wavenumber range of 4000–400 cm^−1^. All the spectra were measured at a spectral resolution of 8 cm^−1^, and 64 scans were taken per sample. The ground-bamboo scrimber powder (2 mg) was mixed with KBr (200 mg) in an agate mortar to form pellets. Tests were repeated three times for each group.

#### 3.2.4. Density and Air-Dried Moisture Content Test

The density and air-dried moisture content tests were carried out according to Chinese standard GB/T 17657-2013. The untreated and treated bamboo scrimber (oil was drained) with the dimensions of 50 (Length) × 50 (Width) × 10 (Thickness) mm^3^ was air-dried in a chamber at a condition of (20 ± 2) °C, (60 ± 5)% relative humidity until the mass was constant, and then the density was determined. After being weighed, the bamboo scrimber was dried to a constant mass at a temperature of (103 ± 2) °C. The oven-dried specimens were immediately cooled in a dryer and weighed at room temperature. Air-dried moisture content was calculated according to the following Equation (1):

H(%) = 100 × (m_0_ − m_1_)/m_1_ (1)

where H represents the air-dried moisture content, m_0_ represents the mass before oven drying, and m_1_ represents the mass after oven drying.

MEE was calculated according to the Equation (2):

MEE (%) = 100 × (H_C_ − H_T_)/H_C_ (2)

where H_C_ represents the air-dried moisture content of untreated group and H_T_ represents the air-dried moisture content of treated group.

#### 3.2.5. Dimensional Stability Test

The 24 h thickness swelling rate, 24 h width swelling rate, and 24 h water absorption rate were carried out according to GB/T 17657-2013. The untreated and treated bamboo scrimbers with dimensions of 150 (Length) × 50 (Width) × 10 (Thickness) mm^3^ were conditioned in a chamber maintained at 20 °C and relative humidity 65% until the mass was constant. Additionally, the samples were immersed in a water tank with a pH of 7 and a temperature of 20 °C. The 24 h thickness swelling rate, 24 h width swelling rate, and 24 h water absorption rate were calculated according to the following Equations (3)–(5):

T (%) = 100 × (t_2_ − t_1_)/t_1_ (3)

B(%) = 100 × (b_2_ − b_1_)/b_1_ (4)

W(%) = 100 × (m_2_ − m_1_)/m_1_ (5)

where T, t_1,_ and t_2_ represent the 24 h thickness swelling rate, the thickness before water absorption, and the thickness after water absorption; B, b_1,_ and b_2_ represent the 24 h width swelling rate, the width before water absorption, and the width after water absorption; and W, m_1,_ and m_2_ represent the water absorption rate, the mass before water absorption, and the mass after water absorption.

RWA was calculated according to the following equation (6):

RWA(%) = (W_C_ − W_T_)/W_C_ (6)

where W_C_ represents the 24 h water absorption rate of untreated group and W_T_ represents the 24 h water absorption rate of treated group.

#### 3.2.6. Mechanical Properties Tests

The MOR and modulus of elasticity (MOE) tests were carried out according to GB/T 17657-2013. Each sample with the dimensions 250 (Length) × 50 (Width) × 10 (Thickness, parallel to the force) mm^3^ was conditioned in a chamber maintained at 20 °C and 65% relative humidity until the weight was constant prior to testing. Then, the three-point bending method was used for the measurement using a universal testing machine (CMT4204, Shenzhen Xinsansi Material Testing Co., Ltd., Shenzhen, China).

#### 3.2.7. Surface Color Measurements

The surface color of the bamboo scrimber samples was measured with a color measurement instrument (CM-5, Konica Minolta, Tokyo, Japan). The test indexes are L*, a*, and b*. The color difference is defined according to the formula.

∆E* = [ (ΔL)^2^ + (Δa*)^2^ + (Δb*)^2^]^1/2^ (7)

where ∆E* is the color difference, ΔL* is the lightness difference, and Δa* and Δb* are the chroma differences. Δ means the differences between the initial and final parameters of the samples after the treatments. L*, a*, and b* are the average values of six positions on each sample.

#### 3.2.8. Contact Angle Measurement

The measurement of the contact angle (wettability) of the untreated and treated samples was performed with a contact angle meter (OCA 40 Micro, Beijing Dongfang Devi Instrument Co., LTD., Beijing, China). The samples with the dimensions of 10 (Thickness) × 50 (Width) × 50 (Length) mm^3^ were taken from the same piece of bamboo scrimber. The surface of the sample was ground with 320 sandpaper, and all samples were kept at standard room temperature (25 °C) and relative humidity (65%). Fluid was distilled water. 10 samples were used for each treatment, and 2 drops per sample were captured. Twenty measurements of contact angles were obtained and the mean value was recorded.

#### 3.2.9. *p*-Test

*p*-test was used to compare the effect of time and temperature on the air-dry density, MOR, MOE, air-dried moisture content, 24 h width swelling rate, 24 h thickness swelling rate, and 24 h water absorption by using SPSS 12.0 with a significance level of *p* = 0.05.

## 4. Conclusions

The effects of hot oil treatment on the density, dimensional stability, mechanical strength, and microscopic chemical and surface properties of bamboo scrimber were determined in this paper. The environmental-scanning electron microscope observation indicated that the glue layer of the bamboo scrimber was not significantly damaged after hot oil treatment, and the mechanical properties did not change significantly at low temperatures. Infrared-spectrum analysis showed a significant decrease in mechanical properties at higher temperatures and longer treatment time for the degradation of hemicellulose. The contact angle and swelling properties test showed that the hot oil treatment improved dimensional stability and reduced the wettability on the surface of the bamboo scrimber. As the *p*-test analysis shows, time and temperature have a significant impact on mechanical strength, swelling properties, water absorption, MEE, and RWA. In general, treatment at 140 °C for 2 h is most effective. In order to further determine the effectiveness of the treatment, it is recommended to test the mold resistance of the bamboo scrimber.

## Figures and Tables

**Table 1:** The color index of bamboo scrimber after hot oil treatment.

Temperature (°C)	Time (h)	L*	a*	b*	ΔE*
Untreated		62.5 (1.3)	9.61(0.7)	26.8(1.4)	-
120	2	52.1 (1.2)	15.9 (1.2)	26.8 (0.9)	14.8 (1.0)
	4	49.1 (1.6)	17.2 (0.5)	35.4 (1.2)	16.8 (0.9)
	6	48.7 (1.9)	16.8 (0.3)	33.5 (1.8)	17.1(1.0)
140	2	47.1 (2.3)	16.7 (1.1)	33.9 (1.5)	18.1 (0.6)
	4	44.3 (1.8)	16.2 (1.3)	33.1 (0.9)	19.5 (1.6)
	6	42.6 (1.5)	16.2 (1.2)	28.8 (0.9)	21.0 (0.6)
160	2	41.3 (1.8)	15.1 (0.3)	27.8 (1.2)	21.9 (0.6)
	4	38.4 (1.3)	13.6 (0.3)	25.5 (1.0)	24.9 (1.2)
	6	36.4 (1.0)	14.1 (0.5)	21.9 (1.1)	27.2 (1.2)
